# Supplementary material for: Genetic and environmental determinants of stressful life events and their overlap with depression and neuroticism
Source: Wellcome Open Res. 2019 Jan 14;3:11. Originally published 2018 Feb 14. [Version 2] doi: 10.12688/wellcomeopenres.13893.2 (PMC6352921; doi:10.12688/wellcomeopenres.13893.2)
Supplement: Peer review history

# Peer review: Referee response for version 2

**DOI:** 10.21956/wellcomeopenres.16405.r34589

**Published:** 2019-01-28

**Authors:** Margaret Gatz

**Affiliations:** 1 Center for Economic and Social Research, University of Southern California, Los Angeles, CA, USA

The additional analyses of the association between independent life events and MDD-PRS and controlling for current psychological distress when looking at the association between stressful life events and MDD make the results more compelling. The paper now makes a useful contribution to sorting out genetic and environmental explanations for the overlap of stressful life events and depression.

I have read this submission. I believe that I have an appropriate level of expertise to confirm that it is of an acceptable scientific standard.

# Peer review: Referee response for version 1

**DOI:** 10.21956/wellcomeopenres.15103.r32922

**Published:** 2018-08-16

**Authors:** Sarah Cohen-Woods

**Affiliations:** 1 School of Psychology, Faculty of Social and Behavioural Sciences, Flinders University, Adelaide, SA, Australia; 2 Flinders Centre for Innovation in Cancer, Flinders University, Adelaide, SA, Australia; 3 Centre for Neuroscience, Flinders University, Adelaide, SA, Australia

This manuscript is very clearly written, with methods well-articulated and described. The authors need to be commended for such an "easy read".

 The authors highlight limitations themselves, in particular with respect to the need to create a "personal" list of life events which inevitably excludes a number of independent stressors. Further highlighted is the restricted power in the mental health GS cohort.

**Minor points:**

 Abstract: where the author writes "were positively correlated with lifetime MDD status..." in results, they would be better positioned to state associated as the statistics presented relate to association primarily.

 Page 6 - reference to Table 4 for MDD-PRS and dependent/independent SLEs - should be Table 3.

**Significant points:**

 Moderation: In the discussion there is a brief mention of one MDD-PRS*life event interaction study. There is more than one publication in the literature looking at this moderation effect (with conflicting findings). Why did the authors not look at moderation in this cohort? (power / issues with correlation)? This should be mentioned / discussed.

 Comorbidity and mortality in MDD: the distinction between dependent and independent SLEs is interesting, particularly within the hypothesis that one may be more correlated with MDD-PRS than the other. Findings in this paper do not support this; it is  worth considering that factors within the independent SLEs may be markers of elevated illness comorbidity / mortality risk in MDD. This could, to some extent, explain some of the correlation seen in these SLEs and MDD.

 Measurement timing:  the measurement of neuroticism is prior to the measurement of SLEs; this needs to be discussed if it has any impact on interpretation.

 Current mood confounds: Did the authors look at impact of current mood (e.g. BDI?) and influence on i) SLE reporting, ii) MDD reporting? Controlling for current mood may be necessary to further disentangle the genetic correlations.

 An interesting addition to the post-GWAS literature.

I have read this submission. I believe that I have an appropriate level of expertise to confirm that it is of an acceptable scientific standard, however I have significant reservations, as outlined above.

# Response

**Published:** 2019-01-09

**Authors:** Toni-Kim Clarke

**Affiliations:** University of Edinburgh, UK

*1. We have changed our abstract to read as the reviewer suggests.*

*2. The references to table 3 and table 4 have been checked and changed where suggested. There was also incorrect referencing of table 5 which has now been updated.*

 3. *We did not analyse the interaction between MDD-PRS and life events in this study as we did not wish to duplicate the efforts of a parallel study of ours which has now been published and covers this topic. (Available here on biorxiv https://www.biorxiv.org/content/early/2018/11/28/479691 and currently in press at Translational Psychiatry).*

 4. Re comorbidity and mortality in MDD: *This is an interesting point raised by the reviewer and we have added the following sentence to the discussion to acknowledge* *this ‘Given that independent SLE may be co-morbid with illnesses and disease which are also co-morbid with MDD, this could explain some of the shared genetic aetiology of these traits’.*

 5. Re measurement timing: *We add the following section to the discussion to acknowledge this*

*‘Finally, neuroticism was measured several years prior to the reporting of the stressful life events. Although this will not change the interpretation of the genetic correlations between neuroticism and SLE the phenotypic association between these traits should be treated with some caution.’*

*6. *Re current mood confounds: *When analysing the genetic correlation between PRS and SLE we controlled for MDD status and found that the genetic correlations remained although they were slightly attenuated and we have stated this in the results section. We perform an additional analysis controlling for current psychological distress assessed using the General Health Questionnaire-28, which was completed at the same time that the participants reported on SLE. This has been added to Table 3 and shows a further attenuation of the association between MDD-PRS and SLE, but a significant association remains for total and dependent SLE.*

# Peer review: Referee response for version 1

**DOI:** 10.21956/wellcomeopenres.15103.r33335

**Published:** 2018-07-24

**Authors:** Lance Rappaport

**Affiliations:** 1 Virginia Institute for Psychiatric and Behavioural Genetics, Virginia Commonwealth University, Richmond, VA, USA; 2 Department of Psychology, University of Windsor, Windsor, ON, Canada

The authors are commended for a clearly articulated example of the information available from so-called post-GWAS genomic analyses. While the sample size is limited regarding the heritability of stressful life events (SLEs), major depressive disorder (MDD) and neuroticism, the present manuscript describes intriguing analyses of the association of genetic risk for MDD and neuroticism with SLEs. The authors demonstrate that, as expected, genetic risk for MDD and neuroticism, assessed using polygenic risk score and linkage disequilibrium (LD) score regression, seem more strongly associated with dependent than independent SLEs.

 As the authors note, limited statistical power hampers the influence of this manuscript, which reports heritability estimates for SLEs, MDD, and neuroticism below those found in larger samples and meta-analyses. However, Tables 3 and 4 present compelling evidence that genetic risk for MDD is associated with both dependent and independent SLEs even after adjusting for depression. These findings encourage future research to examine the role of SLEs in the development of MDD rather than as solely a consequence of the disorder. That said, greater detail is needed regarding the timing of self-reported major depressive episodes. Whether SLEs occur following a major depressive episode would not gravely undermine the present findings, but this detail would aid readers’ interpretation of the present study. Additionally, if the data permits, I recommend that the authors adjust for depression severity rather than the binary diagnosis.

 Finally, the authors contrast associations among MDD, neuroticism, and dependent and independent SLEs. Statistical approaches, including bootstrapping procedures, permit estimating confidence intervals on parameters reported in the present manuscript. These should be added to substantiate comparisons, such as the association of the PRS for MDD with dependent and independent SLEs prior to and following adjustment for MDD.

I have read this submission. I believe that I have an appropriate level of expertise to confirm that it is of an acceptable scientific standard, however I have significant reservations, as outlined above.

# Response

**Published:** 2019-01-09

**Authors:** Toni-Kim Clarke

**Affiliations:** University of Edinburgh, UK

1. Re timing of events and current mood

*The MDD case status represents lifetime depression and in most cases would precede the SLE as these are only past 6 month events. In order to try to control for the current mood we have performed an additional analysis controlling for General Health Questionnaire (GHQ) scores. This questionnaire was answered at the same time as participants reported their SLE and represents current psychological distress. The adjustment attenuates the association between MDD-PRS and SLE somewhat but the association remains significant for total and dependent life events. An additional row has been added to table 3 to add these analyses.*

 2. Re comparing effect sizes for associations after adjustment of MDD

*The PRS and SLE scores were scaled to have a mean of 0 and a standard deviation of 1 prior to regression analyses. The reported beta are therefore standardized and should be comparable across the different traits and post/ prior MDD/GHQ adjustment.*

# Peer review: Referee response for version 1

**DOI:** 10.21956/wellcomeopenres.15103.r33334

**Published:** 2018-07-02

**Authors:** Margaret Gatz

**Affiliations:** 1 Center for Economic and Social Research, University of Southern California, Los Angeles, CA, USA

The authors build a persuasive case why it is important to sort out genetic and environmental explanations for overlaps of stressful life events, depression, and neuroticism. Phenotypically, the authors show that lifetime major depressive disorder (MDD) is associated with neuroticism, stressful life events (SLEs) over the past 6 months are associated with MDD, and neuroticism is associated with SLEs. Following a usual practice in stressful life events research, the authors distinguish between dependent events, which are nominally under the individual’s control, and independent events, whose occurrence are beyond the individual’s control. The association with MDD holds for both types of events.

 The sample under study was from Generation Scotland, including nuclear families, couples, and siblings. Therefore, for the genetic analyses, the authors create a category that they call personal life events. These are events extracted from the longer list of SLEs where the events would not be shared by members of the family. This step was essential to assure that correlations between family members did not simply reflect shared events, but rather, their shared genetic relationship. MDD was assessed using the CIDI-SF interview that ascertains lifetime MDD status, age of first onset, and whether there were subsequent episodes. Neuroticism was assessed at initial contact; MDD and SLEs at re-contact.

 In the genetic analyses, using Genome-wide Complex Trait Analysis (GCTA), the authors report heritability = .13 for personal life events, and heritability = .32 for neuroticism, while heritability was not significant for MDD. In polygenic risk score (PRS) association analyses, the authors report that those with higher PRS _MDD _reported more SLE, both dependent and independent, even controlling for occurrence of MDD. The genetic correlation calculated with LD regression was .60 between MDD and dependent SLE, and .21 between MDD and independent SLE.  In turn, higher PRS _Neuroticism_ was not significantly predictive of SLE, once controlling for neuroticism. The genetic correlations between neuroticism and dependent and independent SLE were .25 and .06, respectively.

 These heritability figures are somewhat lower than estimated heritability from twin studies, as is typically found. Indeed, MDD is generally considered substantially heritable. Previous Generation Scotland work, not cited in this report, found pedigree-based heritability of MDD to be between .28 and .44. Twin studies similarly place the estimated heritability of MDD at .37 (95% CI, .31 to .42). Although not the main focus of the current report, the discrepancy between twin and family-based estimates and GCTA is puzzling with regard to our understanding of MDD.

 The finding of shared heritability between SLE and MDD has the important implication that life events should not be regarded as simply environmental risk factors for depression. The further implication is that common genetic effects may explain why personality plays a role in reporting more life events.

 There are two substantial limitations to the study: One stems from the need to use personal life events for the genetic analyses. The personal life events were largely dependent life events, that is, events to whose occurrence the individual likely contributes, e.g., marital separation, being sacked from one’s job. Independent life events were largely excluded, because they are predominantly events that happen to close family members and therefore affect the individual, but are not under the individual’s own control, such as death of a spouse or child. These latter are also two of the life events typically deemed most disruptive. Dependent life events are more likely to be correlated with personal characteristics such as neuroticism or depressive tendencies, as was found in the present study. Similarly, heritability of dependent events is expected to be higher than heritability of independent events. Unpacking the association between independent life events and MDD is potentially the question of most interest. 

 Another issue with the life events questionnaire is its timing. The List of Threatening Life Experiences as developed by Brugha and colleagues (1985; 1990) asked about life events that occurred in the six months preceding onset of a psychiatric problem. In the present study, it is not clear that the interview to assess MDD captured age at onset of most recent episode of depression, including whether it was within the last 6 months and thus after the life events had occurred. It seems probable that most episodes of MDD preceded the life events reported on the questionnaire and used in these analyses, and therefore, the reported life events cannot be considered a risk factor for MDD. It is also unknown whether respondents actually were depressed (or not) when completing the life events instrument, and therefore the extent to which being depressed in itself may inflate the reporting of life events. Finally, the mean number of total SLE was 1.14 for cases and 0.83 for controls, so it is also likely that a large number of cases and controls experienced no life events, personal or otherwise, in the last 6 months, potentially affecting observed variances and covariances.

 The authors give some consideration to the former limitation, although not in its fullness, and not to the latter limitation at all.

I have read this submission. I believe that I have an appropriate level of expertise to confirm that it is of an acceptable scientific standard, however I have significant reservations, as outlined above.

# Response

**Published:** 2019-01-09

**Authors:** Toni-Kim Clarke

**Affiliations:** University of Edinburgh, UK

*We agree with the reviewer that the association between independent life events and MDD is most interesting; however, as we are estimating heritability using family members, and family members will tend to endorse the same independent life events (e.g. death of a family member), the heritability of independent life event will be inflated and so we had to restrict the life events to those which are personal to the individual. We did look at the genetic correlation between independent life events and MDD using LD score regression and polygenic risk scores and show that there is genetic overlap between the two.*

*The timing of the life events and the MDD is an issue as the rightly reviewer states. To address this add an additional analysis to control for current mood when testing the association of MDD-PRS with life events. We control for current psychological distress which was assessed using the General Health Questionnaire-28 at the same time as individuals reported their SLE. The associations between MDD-PRS and total and dependent SLE remain significant after controlling for current mood however the association with independent SLE becomes non-significant (p=0.052). We have added the results to table 3 and add the following to the results section:*

*“Also, the mean number of SLE experienced by both cases and controls was low and therefore may have contributed to the reduced power to detect the heritability of these traits.”*

*“After controlling for current psychological distress (GHQ score) the association with total life events was attenuated further (β=0.041) but remained significant. The association between polygenic risk for MDD and independent life events was no longer significant after controlling for current psychological distress.”*
